# Effect of all-trans retinoic acid on casein and fatty acid synthesis in MAC-T cells

**DOI:** 10.5713/ajas.19.0315

**Published:** 2019-08-23

**Authors:** Xian-Dong Liao, Chang-Hai Zhou, Jing Zhang, Jing-Lin Shen, Ya-Jing Wang, Yong-Cheng Jin, Sheng-Li Li

**Affiliations:** 1 Department of Animal Science, College of Animal Science, Jilin University, Changchun 130062, China; 2 State Key Laboratory of Animal Nutrition, Beijing Engineering Technology Research Center of Raw Milk Quality and Safety Control, College of Animal Science and Technology, China Agricultural University, Beijing 100193, China

**Keywords:** All-trans Retinoic Acid, Casein, Fatty Acid, mRNA Expression, MAC-T Cells

## Abstract

**Objective:**

Caseins and fatty acids of milk are synthesized and secreted by the epithelial cells of the mammary gland. All-trans retinoic acid (ATRA), an active metabolite of vitamin A, has been shown to promote mammary development. This study was conducted to determine the effect of ATRA on casein synthesis and fatty acid composition in MAC-T cells.

**Methods:**

MAC-T cells were allowed to differentiate for 4 d, treated with ATRA (0, 1.0, 1.5, and 2.0 μM), and incubated for 3 d. We analyzed the fatty acid composition, the mRNA expression of casein and fatty acid synthesis-related genes, and the phosphorylation of casein synthesis-related proteins of MAC-T cells by gas chromatography, quantitative polymerase chain reaction, and western blotting, respectively.

**Results:**

In MAC-T cells, ATRA increased the mRNA levels of α_S1_-casein and β-casein, janus kinase 2 (JAK2) and E74-like factor 5 of the signal transducer and activator of transcription 5 β (STAT5-β) pathway, ribosomal protein S6 kinase beta-1 (S6K1) and eukaryotic translation initiation factor 4E binding protein 1 of the mammalian target of rapamycin (mTOR) pathway, inhibited the mRNA expression of phosphoinositide 3-kinase and eukaryotic initiation factor 4E of the mTOR pathway, and promoted the phosphorylation of STAT5-β and S6K1 proteins. Additionally, ATRA increased the de novo synthesis of fatty acids, reduced the content of long-chain fatty acids, the ratio of monounsaturated fatty acids to saturated fatty acids (SFA), the ratio of polyunsaturated fatty acids (PUFA) to SFA, and the ratio of ω-6 to ω-3 PUFA. The mRNA levels of acetyl-CoA carboxylase 1, fatty acid synthase, lipoprotein lipase, stearoyl-CoA desaturase, peroxisome proliferator-activated receptor gamma, and sterol regulatory element-binding protein 1 (SREBP1) were enhanced by ATRA.

**Conclusion:**

ATRA promotes the synthesis of casein by regulating JAK2/STAT5 pathway and downstream mTOR signaling pathway, and it improves the fatty acid composition of MAC-T cells by regulating SREBP1-related genes.

## INTRODUCTION

Milk is known as "nature’s perfect food". It is a rich source of energy and provides high-quality proteins, minerals, and vitamins. Proteins such as casein, β-lactoglobulin, and α-lactalbumin and almost all short- and medium-chain fatty acids (SCFAs and MCFAs) in milk are synthesized by the mammary epithelial cells. Proliferation and differentiation of the mammary epithelial cells and the process of lactation are regulated by hormones such as prolactin, glucocorticoid, and insulin [1–3]. Recent studies have shown that the expression of casein genes requires a combination of prolactin and hydrocortisone [4]. Additionally, insulin induces increased level of expression of casein and fatty acid synthesis-related genes [1]. Casein synthesis in the mammary gland is regulated by the Janus kinase 2/signal transducer and activator of transcription 5 (JAK2/STAT5) pathway and phosphoinositide 3-kinases/RAC-alpha serine/threonine-protein kinase/mammalian target of rapamycin (PI3K/AKT1/mTOR) pathway [5,6]. Fatty acid synthesis is regulated by peroxisome proliferator-activated receptor gamma (PPARγ) and sterol regulatory element binding protein families (SREBPs) [7], and the expression of fatty acid synthesis-related genes such as fatty acid synthase (*FASN*) and acetyl-coA carboxylase 1 (*ACACA*) is the best indicator of fatty acid synthesis [8].

In addition to hormones and cytokines, nutrients are important for the regulation of casein expression and fatty acid synthesis. Studies have shown that amino acids, fatty acids, minerals, and other nutrients regulate the expression of casein and milk fatty acid synthesis-related genes in mammary epithelial cells via the mTOR signaling pathway [9,10]. Puvogel’s study has shown that vitamin A, a necessary nutrient, enhances lactation performance by promoting the secretion of insulin-like growth factor [11]. All-trans retinoic acid (ATRA) is an active metabolite of vitamin A in the body, which plays a critical role in the initiation of mammary gland development in the embryo [12]. Additionally, ATRA induces the differentiation of cells and acts synergistically with prolactin to increase specific casein gene expression in MAC-T cells [13]. However, there are very few reports on the role of ATRA in regulating casein and milk fatty acid syntheses in dairy cows.

The bovine mammary epithelial cell line MAC-T is a model used to study the synthesis and secretion of caseins and milk fatty acids, and the biosynthesis ability of the cells indicates the lactation performance of the cow’s mammary gland. Moreover, MAC-T cells uniformly differentiate and secrete casein proteins and milk fatty acids in response to hydrocortisone, insulin, and prolactin. Therefore, in this study, MAC-T cells were used to study the effects of ATRA on casein and milk fatty acid synthesis.

A previous study on mouse mammary epithelial cells showed that ATRA inhibited differentiation at 10 μM and 1 μM concentrations and induced differentiation at 0.1 μM and 0.01 μM concentrations [14]. Liu et al [15] found that 4-oxo-ROL, one of the metabolites of vitamin A, and retinoic acid at 1 μM concentration inhibited cell proliferation by regulating short-chain dehydrogenase/reductase 1, ATP-binding cassette G1, ras-related C3 botulinum toxin substrate, and adrenergic receptorβ2 gene expression in normal human breast epithelial cells. However, Lee et al [13] revealed that ATRA at 1 μM concentration increased differentiation and casein mRNA expression in MAC-T cells in association with prolactin. Additionally, ATRA induced the expression of κ-casein in P19 embryonal carcinoma cells at 1 μM concentration during neural differentiation [16]. In the current study, we observed a concentration-dependent effect of ATRA in MAC-T cells; ATRA at concentrations ≤2.0 μM promoted the proliferation of MAC-T cells, while at higher concentration inhibited. Therefore in this study, we used 1.0, 1.5, and 2.0 μM ATRA to evaluate the effect on the mRNA levels of casein and fat synthesis-related genes and milk fat composition of differentiated MAC-T cells.

## MATERIALS AND METHODS

### Cell culture

MAC-T cells were kindly provided by Professor Hong-Gu Lee (Konkuk University, Seoul, South Korea). The cells were grown in Dulbecco’s modified eagle’s medium (DMEM)/high-glucose medium (HyClone, Logan, UT, USA) containing 10% fetal bovine serum (Gibco, Gaithersburg, MD, USA), 1% penicillin-streptomycin (HyClone, USA), 5 μg/mL insulin (Sigma-Aldrich, St. Louis, MO, USA), and 1 μg/mL hydrocortisone (Sigma-Aldrich, USA) in a 37°C incubator with an atmosphere of 5% CO_2_ and air. After the cells reached confluence in 100 mm dishes or 6-well plates, differentiation was induced by replacing the medium with DMEM high glucose medium containing 5% fetal bovine serum, 5 μg/mL insulin, 1 μg/mL hydrocortisone, 5 μg/mL prolactin (Sigma-Aldrich, USA), and 1% penicillin-streptomycin for 4 d as previously described [17]. ATRA (0, 1.0, 1.5, or 2.0 μM) was added to the culture medium and the cells were incubated for another 3 d. Stock solution (10 mM) was prepared by dissolving ATRA (Sigma-Aldrich, USA) in dimethylsulphoxide (DMSO, Sigma-Aldrich, USA), and further diluted with sterile purified water in dark. DMSO (0.02%) was used as the vehicle control. During differentiation, the induction medium was replaced every day and three replicates were maintained for each treatment.

### Quantitative polymerase chain reaction analysis

Cells cultured in 6-well plates were washed twice with phosphate-buffered saline (PBS) and scraped using a cell scraper after adding 1 mL of TRIzol reagent (Thermo Scientific, Waltham, MA, USA). The cell lysates were washed several times by passing through a pipette, transferred to 1.5 mL eppendorf tubes, and stored at −80°C until further analysis. For transcriptional analysis, total RNA was extracted from harvested MAC-T cells using TRIzol reagent according to the manufacturer’s instructions. The concentration, purity, and integrity of the total RNA samples were measured using a NanoDrop 2000 spectrophotometer (Thermo Scientific, USA).

cDNA was synthesized in a Life ECO gene amplification instrument (BIOER, Hangzhou, China) using the HiFi Script cDNA Synthesis Kit (CWBIO, Beijing, China) according to the manufacturer’s instructions in a reaction volume of 20 μL containing 4 μL of dNTP mix, 2 μL of primer mix, 4 μL of 5× reverse transcription buffer, 2 μL of DTT, 1 μL of HiFi Script, and 1 μg of RNA template. The cDNA was synthesized by incubating at 42°C for 15 min followed by 85°C for 5 min.

Quantitative polymerase chain reaction (qPCR) was performed in a Stratagene Mx3005P system (Agilent Technologies, Santa Clara, CA, USA) using UltraSYBR mixture (CWBIO, China) according to the manufacturer’s instructions (10 μL of 2× UltraSYBR mixture, 0.2 μM of forward primer, 0.2 μM of reverse primer, and 10 ng of template DNA in a 20 μL reaction volume). The following PCR program was used: an initial denaturation at 95°C for 10 min followed by 45 cycles of amplification at 95°C for 10 s, annealing at 60°C for 30 s, and extension at 72°C for 32 s. The genes and their specific PCR primers are listed in Table 1. β-Actin was used as a reference gene. Relative gene expression levels for different samples were quantified using the 2^−ΔΔCT^ method.

### Western blotting

Cells cultured in 6-well plates were washed twice with PBS and mixed with 0.5 mL of radio immunoprecipitation assay lysis buffer (Sigma-Aldrich, USA) containing 1 × protease inhibitor cocktail (Amresco Biochemicals and Life Science, Solon, OH, USA). The mixture was incubated for 30 min on ice, transferred to 1.5 mL eppendorf tubes, and centrifuged at 12,000×g for 30 min at 4°C, and the supernatant was collected for western blotting. Protein concentrations were measured using bicinchoninic acid protein assay (Pierce, Rockford, IL, USA). The proteins (30 μg) were separated by sodium dodecyl sulfate polyacrylamide gel electrophoresis (10%, w/v) and transferred to nitrocellulose membrane (Millipore Corp, Billerica, MA, USA). The membranes were blocked for 3 h with 5% skim milk in Tris-buffered saline-Tween (TBST) buffer (20 mM Tris-HCl, pH 7.6; 137 mM NaCl; and 0.01% Tween 20). The membranes were then incubated overnight at 4°C with primary antibodies: rabbit anti-bovine β-actin polyclonal antibody (1:2,000; Bioss, Beijing, China), rabbit anti-bovine phospho-STAT5-β polyclonal antibody (1:500; Bioss, China), rabbit anti-bovine phospho-mTOR polyclonal antibody (1:500; Bioss, China), or rabbit anti-bovine phospho-S6K1 polyclonal antibody (1:500; Bioss, China). The membranes were washed 3 times with 1× TBST and incubated with goat anti-rabbit immunoglobulin G antibody (1:2,000; Bioss, USA) for 3 h at 4°C. The membranes were incubated with ECL western blotting substrate (Thermo Scientific, USA), and visualized using chemiluminescence imaging system (Tanon, Shanghai, China). The protein bands were quantified using ImageJ 1.52a software (National Institutes of Health, Bethesda, MD, USA). Protein levels were normalized by comparing signals with β-actin on the same membrane.

### Cellular fatty acid methyl ester analysis

Cells cultured in 100 mm plates were washed twice with PBS; total fatty acids from the cells were extracted using Folch’s solution (chloroform/methanol solution; 2:1, vol/vol) and converted to methyl esters using methyl esterification solution (BF3/methanol/hexane; 4.9:75.1:20, vol/vol). Chromatographic analysis was performed using gas chromatography system (GC-7980, Techcomp, Shanghai, China) with silica capillary column (SP^T^-2560, 100 m×0.25 mm×0.20 μm film thickness; Supelco, Bellefonte, PA, USA). The oven was set at a temperature of 70°C for 1 min, increased to 100°C at a rate of 5°C/min, maintained at 100°C for 2 min, increased to 175°C at a rate of 10°C/min, maintained at 175°C for 40 min, further increased to 225°C at a rate of 5°C/min, and maintained at 225°C for 40 min. Sample or standard mixture of fatty acid methyl esters (1 μL; FAMEs; Supelco 37-component FAME Mix, Supelco, USA) was injected into the column, carried by nitrogen, and detected by hydrogen flame ionization detector. The FAME components of sample were determined by comparing retention time peak with the appropriate FAME standards. Individual fatty acids were expressed as a weight percentage of the total fatty acids analyzed.

### Statistical analysis

The experimental data were analyzed using SPSS 21.0 (SPSS Inc., Chicago, IL, USA) and expressed as mean±standard deviation. Two-treatment effects were evaluated using an independent sample t-test or one-way analysis of variance followed by the Duncan multiple range test. A value of p<0.05 was considered statistically significant.

## RESULTS

### Effect of all-trans retinoic acid on mRNA expression of casein in MAC-T cells

**Figure 1:**
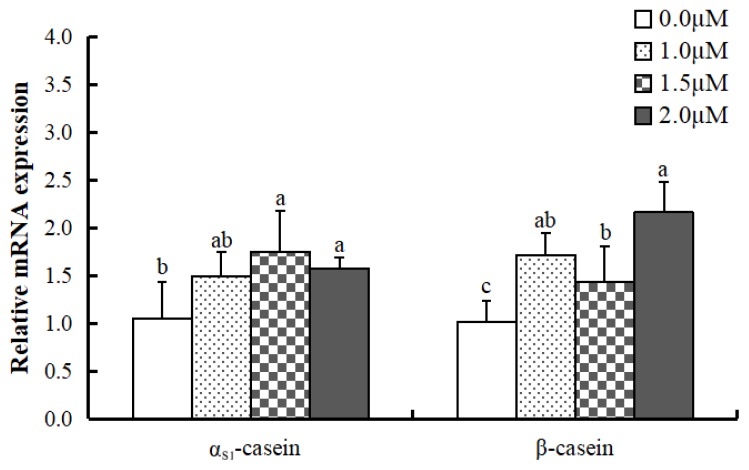
Casein mRNA expression levels in differentiated MAC-T cells treated with all-trans retinoic acid. Data are mean±standard deviation (n = 3). ^a–c^ mRNA levels of the same gene with different superscript letters are significantly different (p<0.05).

The study determined the effect of ATRA on mRNA expression of α_S1_-casein and β-casein in MAC-T cells by qPCR (Figure 1). α_S1_-Casein mRNA expression was significantly higher in cells treated with 1.5 and 2.0 μM ATRA (p<0.05) compared to control. β-Casein mRNA expression was significantly higher in cells treated with ATRA at all concentrations compared to control (p<0.05), and 1.0 or 2.0 μM ATRA was the most effective in promoting β-casein mRNA expression.

### Effect of ATRA on mRNA expression of casein synthesis-related genes and phosphorylation of casein synthesis-related proteins in MAC-T cells

**Figure 2:**
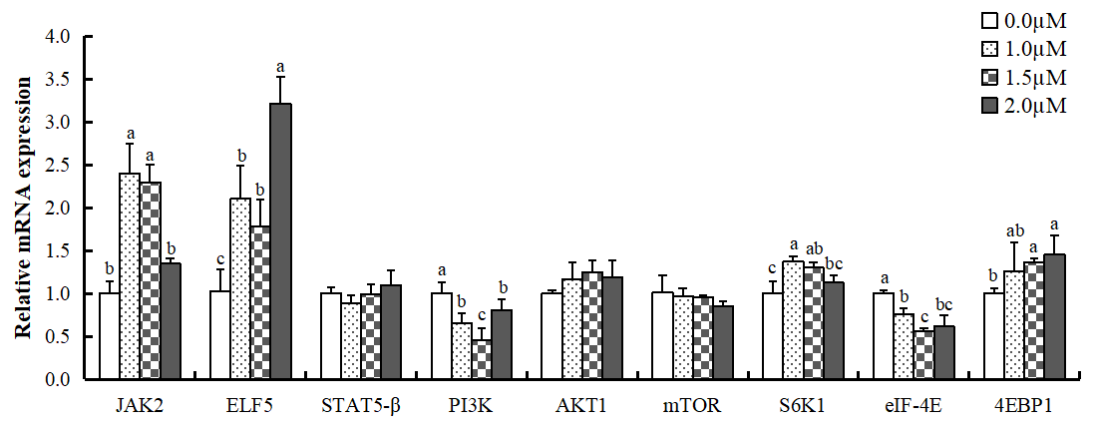
mRNA expression levels of casein synthesis-related genes in differentiated MAC-T cells treated with all-trans retinoic acid. JAK2, janus kinase 2; ELF5, E74-like factor 5; STAT5-β, signal transducer and activator of transcription 5 β; PI3K, phosphoinositide 3-kinase; AKT1, RAC-alpha serine/threonine-protein kinase; mTOR, mammalian target of rapamycin; S6K1, S6 kinase beta-1; eIF4E, eukaryotic initiation factor 4E; 4EBP1, 4E binding protein 1. Data are mean±standard deviation (n = 3). ^a–c^ mRNA levels of the same gene with different superscript letters are significantly different (p<0.05).

**Figure 3:**
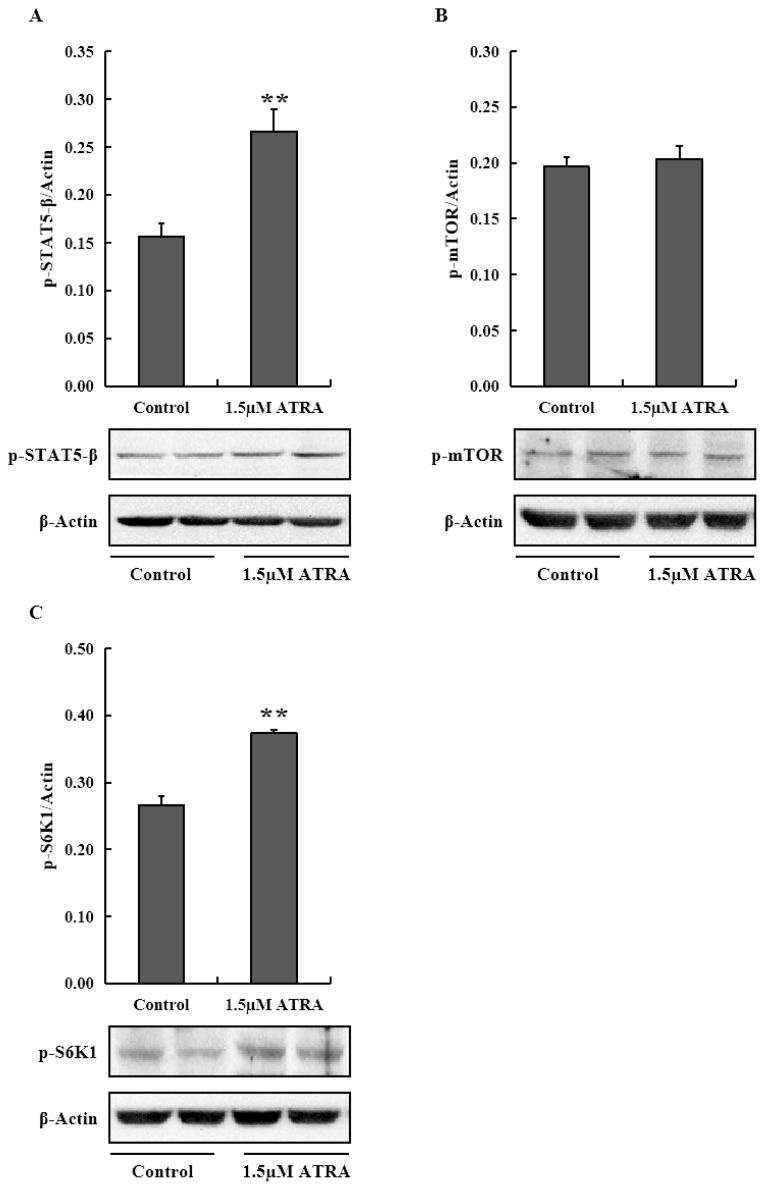
Protein levels of phospho-STAT5-β (A), phospho-mTOR (B), and phospho-S6K1 (C) in differentiated MAC-T cells treated with ATRA. STAT5-β, signal transducer and activator of transcription 5 β; mTOR, mammalian target of rapamycin; S6K1, S6 kinase beta-1; ATRA, all-trans retinoic acid. Data are mean±standard deviation (n = 3). ** Astersik indicates significant difference comparing with control (p<0.01).

To examine the role of JAK2/STAT5 and mTOR signaling pathways in promoting casein synthesis by ATRA, we analyzed the mRNA levels of genes in the two signaling pathways in MAC-T cells (Figure 2). The mRNA level of JAK2 was significantly higher in cells treated with 1.0 and 1.5 μM ATRA compared to control (p<0.05), and the mRNA level of E74-like factor 5 (ELF5) in cells treated with ATRA was significantly more than control (p<0.05), and 2.0 μM ATRA was the most effective in promoting ELF5 mRNA expression. STAT5-β mRNA showed no significant difference among groups; however, the level of phosphorylated STAT5-β protein was significantly higher in cells treated with 1.5 μM ATRA compared to control (p<0.01; Figure 3A).

PI3K mRNA expression was inhibited by ATRA (p<0.05; Figure 2), especially at 1.5 μM concentration; however, AKT1 and mTOR mRNA levels and phosphorylated mTOR protein level were not affected by ATRA at 1.5 μM concentration (Figure 3B). With respect to downstream genes of mTOR signaling pathway, ribosomal protein S6 kinase beta-1 (S6K1) mRNA level was upregulated in cells treated with 1.0 and 1.5 μM ATRA (p<0.05), while eukaryotic translation initiation factor 4E binding protein 1 (4EBP1) mRNA level was higher in cells treated with 1.5 and 2.0 μM ATRA (p<0.05). Eukaryotic translation initiation factor 4E (eIF4E) mRNA expression was inhibited by ATRA at all concentrations (p<0.05). Moreover, the level of phospho-S6K1 protein was significantly higher in cells treated with 1.5 μM ATRA compared to control (p<0.01; Figure 3C).

### Effect of all-trans retinoic acid on fatty acid composition of MAC-T cells

To determine the effect of ATRA on fatty acid composition of differentiated MAC-T cells, gas chromatography was performed, and the weight percentage of fatty acids is presented in Table 2. Analysis revealed a higher proportion of lauric acid (C12:0), myristoleic acid (C14:1), γ-linolenic acid (C18:3c, ω-6), and eicosapentaenoic acid (EPA, C20:5c, ω-3) in cells treated with 1.0 μM ATRA (p<0.05). Palmitic acid (C16:0) and cis-15-tetracosaenoic acid (C24:1) significantly increased when treated with 2.0 μM ATRA (p<0.05). The secretion of butyric acid (C4:0), caproic acid (C6:0), octanoic acid (C8:0), cis-11-eicosenoic acid (C20:1), and lignoceric acid (C24:0) was higher in 1.5 and 2.0 μM ATRA treated cells (p<0.05). Myristic acid (C14:0), pentadecanoic acid (C15:0), heptadecanoic acid (C17:0), cis-10-heptadecenoic acid (C17:1), stearic acid (C18:0), erucic acid (C22:1), and docosahexaenoic acid (DHA, C22:6c, ω-3) were significantly more at all concentrations than control (p<0.05). Meanwhile, the secretion of heneicosanoic acid (C21:0) was significantly reduced by 2.0 μM ATRA (p<0.05), behenic acid (C22:0) by 1.5 and 2.0 μM ATRA (p<0.05), and linoleic acid (C18:2c, ω-6) by all concentrations (p<0.05).

Moreover, the comprehensive analysis of fatty acid composition is given in Table 3. The proportion of SCFAs and MCFAs was significantly higher while LCFAs was significantly lower in cells treated with 1.5 and 2.0 μM ATRA compared to control (p<0.01). The proportion of total saturated fatty acids (SFAs; p<0.01) and monounsaturated fatty acids (MUFAs; p<0.05) in MAC-T cells significantly increased when treated with ATRA at all concentrations. Meanwhile, polyunsaturated fatty acids (PUFAs) were significantly lowered by ATRA at all concentrations (p<0.01). The ratio of MUFA/SFA was significantly less in cells treated with 1.5 μM (p<0.05); however, there was no significant difference at other concentrations compared to control, and PUFA/SFA was significantly less at all concentrations of ATRA (p<0.01). Additionally, ω-6 unsaturated fatty acids reduced while ω-3 unsaturated fatty acids increased in cells at all concentrations of ATRA (p< 0.01), and the ratio of ω-6/ω-3 significantly decreased at all concentrations of ATRA (p<0.01).

### Effect of all-trans retinoic acid on mRNA expression of fatty acid synthesis-related genes in MAC-T cells

**Figure 4:**
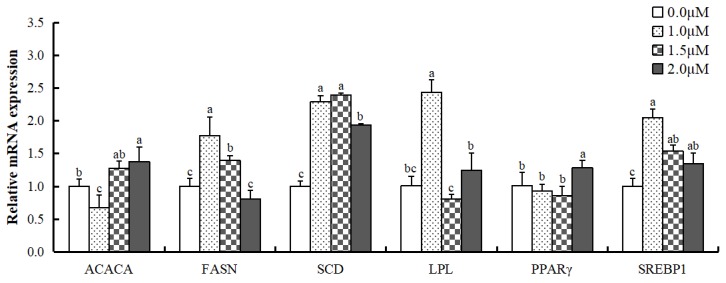
mRNA expression levels of fatty acid synthesis-related genes in differentiated MAC-T cells treated with all-trans retinoic acid. ACACA, acetyl-coA carboxylase 1; FASN, fatty acid synthase; SCD, stearoyl-CoA desaturase; LPL, lipoprotein lipase; PPARγ, peroxisome proliferator-activated receptor gamma; SREBP1, sterol regulatory element-binding protein 1. Data are mean±standard deviation (n = 3). ^a–c^ mRNA levels of the same gene with different superscript letters are significantly different (p<0.05).

Experiments revealed the effect of ATRA on carbon chain shortening, desaturation, and ω-6 to ω-3 fatty acid conversion. Therefore, we further examined the changes in mRNA levels of genes related to fatty acid synthesis (Figure 4). ACACA mRNA level was upregulated when treated with 2.0 μM ATRA (p<0.05); however, the expression was inhibited by 1.0 μM ATRA (p<0.05). FASN mRNA level was significantly more in cells treated with 1.0 and 1.5 μM ATRA compared to control (p<0.05), and did not show any significant difference when treated with 2.0 μM ATRA. Stearoyl-CoA desaturase (SCD) mRNA level was significantly more than control when treated with ATRA at all concentrations. Lipoprotein lipase (LPL) mRNA expression was significantly higher in cells treated with 1.0 μM ATRA (p<0.05); however, showed no significant difference at other concentrations compared to control, and PPARγ mRNA expression was significantly higher in cells treated with 2.0 μM ATRA compared to control (p< 0.05). SREBP1 mRNA expression increased in cells treated with ATRA at all concentrations (p<0.05).

## DISCUSSION

The major milk protein is casein, and α_S1_-casein and β-casein are the main components reflecting casein synthesis. Casein synthesis is mainly regulated by two signaling pathways, namely JAK2/STAT5 and mTOR signaling pathways [18]. STAT5-β is one of the most important transcription factors controlling the expression of casein genes. The binding of cytokines to their corresponding receptors activates JAK. JAK further activates STAT5 dimer proteins in the nucleus, thereby promoting the expression of casein genes. In this pathway, ELF5, a co-regulator of the STAT5-β signaling pathway, plays an important role in regulating casein synthesis in mice [19]. In this study, ATRA promoted mRNA expression of α_S1_-casein and β-casein, increased the mRNA levels of JAK2 and ELF5 of the JAK2/STAT5-β signaling pathway, and upregulated the protein level of phospho-STAT5-β. Meanwhile, STAT5-β mRNA showed no significant difference. These findings suggested that ATRA increases JAK2 and ELF5 expression, activates STAT5-β by phosphorylation, and eventually promotes casein expression. Reichenstein et al [20] reported that the knockout of *STAT5-β* gene in mouse mammary gland impaired the expression of whey acidic protein and lactoalbumin; however, there was no effect on β-casein. Chiba et al [21] discovered that serotonin suppresses β-casein expression via inhibition of STAT5 protein phosphorylation in human mammary epithelial cells. These similar results suggested that STAT5-β regulates casein expression via phosphorylation at the protein level.

The mTOR is a master regulator of RNA translation. PI3K transmits growth signals to AKT1 to phosphorylate proline-rich Akt substrate of 40 kDa (PRAS40), and the activation of PRAS40 decreases its suppressive effect on mTOR. Activated mTOR activates S6K1 and dissociates the dimer formed by eIF4E and 4EBP1. Dissociated eIF4E ultimately participates in the formation of translation initiation complex. In the study by Peng et al [22], addition of glucose enhanced protein synthesis by increasing ATP, and promoted the expression of the mTOR pathway indirectly in bovine mammary epithelial cells. Nan et al [23] reported that lysine and methionine promoted mRNA levels of casein genes (α_S1_-casein, β-casein) by increasing the mRNA levels of JAK2, STAT5-β, ELF5, and mTOR, and by reducing the mRNA levels of eIF4E and 4EBP1 in bovine mammary cells. Zhang et al [24] observed significant increase in the mRNA levels of β-casein, AKT1, mTOR, and S6K1 in bovine mammary epithelial cells treated with methionine. In summary, different factors regulate casein synthesis, including glucose and amino acids, and these factors are related to mTOR pathway, which indicates the importance of mTOR pathway in casein synthesis. However, in our study, PI3K mRNA expression was inhibited by ATRA, and AKT1 and mTOR mRNA levels and mTOR phosphorylation were not affected by ATRA. These results indicated that the promotion of α_S1_-casein and β-casein mRNA by ATRA is not associated with the upstream of PI3K/AKT/mTOR signaling pathway. Meanwhile, S6K1 and 4EBP1 mRNA levels were higher in cells treated with ATRA, and eIF4E mRNA expression was inhibited by ATRA, which is consistent with the previous finding. Taken collectively, there must be an alternative pathway including *S6K1*, *4EBP1*, and *eIF4E* genes, which regulates the effect of ATRA on the expression of α_S1_-casein and β-casein genes.

Fatty acids are classified into SFAs, MUFAs, and PUFAs. Dietary intake of SFAs increases the level of cholesterol in humans, which are not conducive to good health. Whereas, PUFAs are helpful to reduce the incidence of human diseases such as cancer, obesity, and cardiovascular disease [25]. Therefore, milk with higher PUFA/SFA ratio is more beneficial to human health. PUFAs are classified into several categories characterized by the presence of a double bond three atoms away from the terminal methyl group. Among these categories, ω-6 and ω-3 PUFAs are essential for the human body. The dynamic balance of ω-6 and ω-3 PUFAs plays an important role in the normal growth and development of the human body. World health organization (WHO) and Food and agriculture organization (FAO) proposed that the appropriate ratio of ω-6/ω-3 PUFAs in the diet is (5–10):1. However, the proportion of ω-6/ω-3 PUFAs in human daily diet is too high and is increasing every year [26] that leads to high incidence of a variety of diseases, including prostate cancer [27]. Comprehensive analysis of the fatty acids revealed an increase in the proportion of SCFAs and MCFAs and a decrease in the proportion of LCFAs on treating the mammary epithelial cells with ATRA. This indicated that ATRA helps improve fatty acid composition and increases its absorption rate. Meanwhile, ATRA significantly increased the proportion of ω-3 PUFAs, especially EPA and DHA, which are associated with blood vessel function and cognitive function, respectively, and reduced the ratio of ω-6/ω-3 from 15 to 5–8, which is in line with the optimal ratio proposed by WHO and FAO. In the concentration range we studied, higher concentration of ATRA had stronger effect on ω-6/ω-3 PUFAs. It indicated that ATRA could effectively ameliorate the unhealthy status of human ω-6 and ω-3 PUFAs intake.

In our study, ATRA improved the proportion of most fatty acids in MAC-T cells and upregulated the mRNA expression of ACACA, FASN, SCD, LPL and SREBP1. Bernard et al [28] reported that the mRNA levels of ACACA and FASN in the mammary gland are positively correlated with their enzymatic activities, and the enzymatic activities of ACACA and FASN are positively correlated with de novo synthesis of fatty acids. The increase in ACACA and FASN mRNA levels indicated that ATRA promotes de novo synthesis of fatty acids in MAC-T cells. On the other hand, SCD is the key enzyme for the synthesis of MUFAs and PUFAs in the mammary gland [29], and it was upregulated by ATRA in MAC-T cells, and hence, MUFA in the cells increased when treated with ATRA. However, the MUFA/SFA ratio decreased. This may be because the increase in SFAs is more than the conversion of SFAs to MUFAs. Bernard et al [28] reported that *LPL* gene is involved in fatty acid uptake and transport, and very low density lipoprotein receptor works in conjunction with LPL to hydrolyze triacylglycerol (TAG) and release fatty acid and glycerol in the ruminant lactating mammary gland. Therefore, ATRA may promote the metabolism of TAG by increasing the mRNA level of LPL to produce more fatty acid.

SREBP1 is a ligand activation receptor that belongs to the nuclear hormone receptor family. As an important transcriptional regulator in the fatty acid synthesis gene network, it can directly regulate target genes such as *ACACA*, *FASN*, and *SCD*. Furthermore, it regulates the transport and synthesis of milk fats, especially the de novo synthesis of SFAs and desaturation of fatty acids [30]. In our study, the mRNA expression level of SREBP1 was recorded the highest at 1.0 μM ATRA, this was consistent with the mRNA levels of FASN, SCD, and LPL, which suggested a link between SREBP1 and these genes. Collectively, this indicated that ATRA may upregulate the mRNA expression of fatty acid synthesis-related gene such as *FASN*, *SCD*, and *LPL* by regulating SREBP1 signaling pathway, and thereby improve the composition of milk fatty acids.

In summary, ATRA promotes the synthesis of casein through regulated JAK2/STAT5 pathway and downstream mTOR signaling pathway. Treatment with ATRA improves the fatty acid composition in MAC-T cells, which increases the de novo synthesis of stearic acid and DHA and reduces the ratio of ω-6/ω-3 fatty acids possibly by regulating the SREBP1 signaling pathway. This particular *in vitro* study implicated that ATRA could improve the quality of dairy products, which provides new ideas for production and application in the dairy industry.

## Figures and Tables

**Table 1:** *Bos taurus* primers used for quantitative polymerase chain reaction

Primer	Accession number	Forward primer (5′-3′)	Reverse primer (5′-3′)	Product size (bp)
β-actin	NM_173979.3	CTCTTCCAGCCTTCCTTCCT	GGGCAGTGATCTCTTTCTGC	178
α_S1_-casein	NM_181029.2	ACTGAGGATCAAGCCATGGAAG	GAATGTGCTTCTGCTCAACACT	100
β-casein	XM_010806178.2	ACCAGCCTCTTCCTCCAACT	GCCTGAATGGGCATATCTCT	124
JAK2	XM_005209981.4	CAAGACCAGATGGATGCCCAG	ACTCGAACTGCTAGGTCTCTGA	103
STAT5-β	NM_174617.4	GAGAACACCCGCAATGATTAC	TCACCGACTCTGCTCCACG	151
ELF5	NM_001024569.1	CATCCGCTCACAAGGTTACTC	CTCGCACAAATTCCCATAGAT	170
PI3K	NM_001206047.1	GTCTGGACCTTCGGATGCTAC	TAAACTCCTCAATGGCTCGGT	213
AKT1	NM_173986.2	GCACAAGCGAGGTGAGTACAT	GCCACGGAGAAGTTGTTGAG	138
mTOR	XM_002694043.6	CGAAGAACCAATTATACCCGC	CATAGCAACCTCAAAGCAGTCC	153
S6K1	NM_205816.1	AATGCTGCTTCTCGTCTTGGA	CAGTTCTTCCCAGTTAATATGTCT	90
EIF4E	NM_174310.3	CCCGCCTACAGAAGAAGAGA	CAGTATCAAACTTAGAGATCAATCG	164
4EBP1	NM_001077893	TCACTAGCCCTACAGGCGAT	AAACTGTGACTCTTCACCGC	101
ACACA	NM_174224.2	GGAGACAAACAGGGACCATTAC	GTGGAAGGAATGCTTGGGAG	187
FASN	NM_001012669.1	GACCTGGGAGGAGTGTAAGC	GCGATAGCGTCCATGAAGTA	198
SCD	NM_173959.4	CCACGTTCTTCATTGATTGC	CAGCCACTCTTGTAGCTTTCC	121
LPL	NM_001075120.1	TCACTTCAACCACAGCAGCA	GATGACGTTGGAGTCCGGTT	127
PPARγ	NM_181024.2	TTCCGTTCCCAAGAGCTGAC	TGGGGATACAGGCTCCACTT	98
SREBP1	NM_001113302.1	CGCTCTTCCATCAATGACA	TTCAGCGATTTGCTTTTGTG	188

JAK2, janus kinase 2; STAT5-β, signal transducer and activator of transcription 5 β; ELF5, E74-like factor 5; PI3K, phosphoinositide 3-kinase; AKT1, RAC-alpha serine/threonine-protein kinase; mTOR, mammalian target of rapamycin; S6K1, S6 kinase beta-1; EIF4E, eukaryotic initiation factor 4E; 4EBP1, 4E binding protein 1; ACACA, acetyl-coA carboxylase 1; FASN, fatty acid synthase; SCD, stearoyl-CoA desaturase; LPL, lipoprotein lipase; PPARγ, peroxisome proliferator-activated receptor gamma; SREBP1, sterol regulatory element-binding protein 1.

**Table 2:** Fatty acid composition of MAC-T cells treated with ATRA

Fatty acid (% total fatty acid)	Concentration of ATRA (μM)				p-value

	0.0	1.0	1.5	2.0	
Butyric acid (C4:0)	0.90±0.09^b^	1.14±0.22^b^	1.97±0.16^a^	1.68±0.25^a^	<0.001
Caproic acid (C6:0)	0.88±0.29^b^	1.24±0.47^ab^	1.62±0.15^a^	1.79±0.11^a^	0.020
Octanoic acid (C8:0)	0.26±0.02^b^	0.35±0.03^b^	0.57±0.07^a^	0.54±0.08^a^	<0.001
Decanoic acid (C10:0)	0.24±0.01	0.24±0.06	0.27±0.05	0.29±0.01	0.433
Lauric acid (C12:0)	0.20±0.02^b^	0.26±0.02^a^	0.23±0.04^ab^	0.10±0.02^c^	<0.001
Myristic acid (C14:0)	1.19±0.24^b^	1.86±0.06^a^	1.90±0.06^a^	1.98±0.08^a^	<0.001
Myristoleic acid (C14:1)	0.12±0.02^b^	0.16±0.01^a^	0.13±0.02^ab^	0.13±0.01^ab^	0.060
Pentadecanoic acid (C15:0)	1.24±0.34^b^	2.76±0.10^a^	2.59±0.24^a^	2.75±0.13^a^	<0.001
Cis-10-pentadecanoic acid (C15:1)	0.44±0.21	0.53±0.21	0.62±0.11	0.60±0.06	0.562
Palmitic acid (C16:0)	27.34±2.35^ab^	27.03±0.80^b^	28.31±0.58^ab^	29.76±0.42^a^	0.116
Palmitoleic acid (C16:1)	2.30±0.84	3.04±0.09	2.99±0.08	3.13±0.06	0.146
Heptadecanoic acid (C17:0)	0.15±0.04^b^	0.43±0.03^a^	0.38±0.07^a^	0.39±0.01^a^	<0.001
Cis-10-heptadecenoic acid (C17:1)	0.44±0.11^b^	0.90±0.01^a^	0.85±0.08^a^	0.89±0.02^a^	<0.001
Stearic acid (C18:0)	7.34±1.23^b^	11.13±0.58^a^	10.86±1.34^a^	10.36±0.20^a^	0.004
Oleic acid (C18:1)	22.94±0.32	23.15±0.82	23.69±0.93	24.28±0.76	0.199
Linolelaidic acid (C18:2t,ω-6)	3.01±0.84	3.44±0.64	3.77±0.04	3.85±0.21	0.286
Linoleic acid (C18:2c,ω-6)	18.65±6.36^a^	3.39±0.59^b^	3.96±0.92^b^	3.15±0.85^b^	0.001
γ-Linolenic acid (C18:3c,ω-6)	2.04±0.27^b^	6.86±0.62^a^	3.57±1.40^b^	3.06±0.84^b^	0.001
α-Linolenic acid (C18:3c,ω-3)	0.85±0.15	0.74±0.10	0.67±0.10	0.68±0.12	0.305
Arachidic acid (C20:0)	0.53±0.14	0.49±0.05	0.61±0.07	0.62±0.08	0.315
Cis-11-eicosenoic acid (C20:1)	0.79±0.08^b^	0.99±0.22^ab^	1.15±0.06^a^	1.11±0.09^a^	0.041
Cis-11,14-eicosadienoic acid (C20:2c,ω-6)	0.02±0.01	0.03±0.02	0.04±0.01	0.03±0.01	0.552
Cis-8,11,14-eicosatrienoic acid (C20:3c,ω-6)	1.07±0.01	0.92±0.19	0.90±0.02	0.96±0.05	0.230
Arachidonic acid (C20:4c,ω-6)	2.35±0.16	2.63±0.12	2.58±0.01	2.61±0.23	0.182
Eicosapentaenoic acid (C20:5c,ω-3)	0.24±0.02^b^	0.28±0.02^a^	0.27±0.03^ab^	0.26±0.02^ab^	0.123
Heneicosanoic acid (C21:0)	1.90±0.07^ab^	2.88±0.79^a^	2.35±0.87^ab^	1.60±0.27^b^	0.127
Behenic acid (C22:0)	0.75±0.16^a^	0.53±0.09^ab^	0.31±0.13^bc^	0.29±0.05^c^	0.004
Erucic acid (C22:1)	0.16±0.05^b^	0.25±0.01^a^	0.23±0.02^a^	0.24±0.03^a^	0.022
Cis-13,16-docosadienoic acid (C22:2c,ω-6)	0.14±0.03	0.14±0.04	0.15±0.03	0.15±0.02	0.985
Docosahexaenoic acid (C22:6c,ω-3)	0.79±0.18^b^	1.36±0.07^a^	1.36±0.01^a^	1.44±0.06^a^	<0.001
Lignoceric acid (C24:0)	0.48±0.06^b^	0.55±0.04^b^	0.85±0.02^a^	0.93±0.04^a^	<0.001
Cis-15-tetracosaenoic acid (C24:1)	0.25±0.02^b^	0.26±0.02^b^	0.27±0.03^b^	0.34±0.02^a^	0.008

Data are mean±standard deviation (n = 3).

ATRA, all-trans retinoic acid.

a–c Values within a row with different superscript letters are significantly different (p<0.05).

**Table 3:** Comprehensive analysis of fatty acid composition of MAC-T cells treated with ATRA

Items1)	Concentration of ATRA (μM)				p-value

	0.0	1.0	1.5	2.0	
SCFAs	0.90±0.09^b^	1.14±0.22^b^	1.97±0.16^a^	1.68±0.25^a^	<0.001
MCFAs	1.58±0.29^b^	2.10±0.54^ab^	2.69±0.19^a^	2.72±0.21^a^	0.010
LCFAs	97.51±0.38^a^	96.76±0.65^a^	95.34±0.21^b^	95.60±0.38^b^	0.001
SFAs	43.40±4.65^b^	50.91±1.07^a^	52.82±1.71^a^	53.09±0.70^a^	0.005
MUFAs	27.43±1.14^b^	29.28±0.57^a^	29.91±0.70^a^	30.72±0.78^a^	0.007
PUFAs	29.16±5.52^a^	19.80±0.64^b^	17.27±1.03^b^	16.19±1.46^b^	0.002
MUFA/SFA	0.64±0.05^a^	0.58±0.02^ab^	0.57±0.03^b^	0.58±0.01^ab^	0.104
PUFA/SFA	0.69±0.20^a^	0.39±0.02^b^	0.33±0.03^b^	0.31±0.03^b^	0.007
ω-6 fatty acids	27.26±5.55^a^	17.39±0.59^b^	14.93±1.12^b^	13.78±1.41^b^	0.002
ω-3 fatty acids	1.88±0.03^b^	2.38±0.16^a^	2.30±0.12^a^	2.38±0.21^a^	0.007
ω-6/ω-3	14.52±3.11^a^	7.31±0.50^b^	6.51±0.80^b^	5.80±0.68^b^	0.001

Data are mean±standard deviation (n = 3).

ATRA, all-trans retinoic acid; SCFAs, short chain fatty acids; MCFAs, medium chain fatty acids; LCFAs; long chain fatty acids; SFAs, saturated fatty acids; MUFAs, monounsaturated fatty acids; PUFAs, polyunsaturated fatty acids.

1) SCFAs: C4:0. MCFAs: sum of C6:0, C8:0, C10:0, and C12:0. LCFAs: sum of fatty acids listed except SCFAs and MCFAs. SFAs: sum of C4:0, C6:0, C8:0, C10:0, C12:0, C14:0, C15:0, C16:0, C17:0, C18:0, C20:0, C21:0, C22:0, and C24:0. MUFAs: sum of C14:1, C15:1, C16:1, C17:1, C18:1, C20:1, C22:1, and C24:1. PUFAs: sum of C18:2t, C18:2c, C18:3c, C18:3c, C20:2c, C20:3c, C20:4c, C22:2c, C20:5c, C22:6c. ω-6: sum of C18:2t, C18:2c, C18:3c, C20:2c, C20:3c, C20:4c, C22:2c. ω-3: sum of C18:3c, C20:5c, C22:6c.

ab Values within a row with different superscript letters are significantly different (p<0.05).
